# Occupational infection and needle stick injury among clinical laboratory workers in Al-Madinah city, Saudi Arabia

**DOI:** 10.1186/s12995-018-0198-5

**Published:** 2018-05-21

**Authors:** Omar F. Khabour, Khalil H. Al Ali, Waleed H. Mahallawi

**Affiliations:** 1 Department of Clinical Laboratory Sciences, Taibah University, Al-Madinah, 41477 Saudi Arabia; 2 Department of Medical Laboratory Sciences, Jordan University of Science and Technology, Irbid, Jordan

**Keywords:** Biosafety, Clinical laboratory, Al-Madinah, Occupational infection, Needlestick

## Abstract

**Background:**

Clinical laboratory workers face biohazard such as needlestick injury and occupational infection on a daily basis. In this study, we examined self-reported frequency of occupational infection and needlestick injury among the clinical laboratory workers in Al- Madinah, Saudi Arabia.

**Methods:**

A total of 234 clinical laboratory workers were recruited from private and government health sectors to answer a self-administered questionnaire that was prepared to achieve the aims of the study.

**Results:**

The results showed that approximately 33% of the sample had an experienced occupational infection while 24% had experienced a needlestick injury. Approximately, 49% reported that they always recap needle after use, whereas 15% reported doing that most of the times. Occupational infection, needlestick injury and recapping needles after use were associated with lack of training on biosafety (*P* < 0.05).

**Conclusion:**

The frequency of occupational infection and needlestick injury among clinical laboratory workers in Al-Madinah is high. Interventions related to biosafety and infection control and the use of needlestick prevention devices might be useful in lowering such frequency.

## Background

Clinical laboratory workers are subjected daily to occupational hazards that include infections from biological samples and contaminated equipment [1]. For example, literature showed that workers at clinical laboratory are at increased risk of acquiring viral infections such as hepatitis viruses (HBV and HCV), human immunodeficiency viruses (HIV), Middle East Respiratory Syndrome (MERS-CoV), and others [2, 3]. In addition, bacterial occupational infection has been shown to be high among clinical laboratory workers and other health care providers [4–6]. For example, in a study that was conducted in the United Kingdom, clinical laboratory workers were at about seven times higher risk of acquiring tuberculosis infection in reference to the general population [7].

One of the major sources of infection among health care professionals is needlestick injuries [8, 9]. According to the literature, needlestick injury is responsible for the majority of hepatitis and HIV infections among health care professionals. In addition, the majority of these infections occur in developing countries [10]. Analysis of needlestick injuries showed that injuries could happen during all steps of needle use procedures [8]. However recapping of the needle, work load and lack of training, and not following safety precautions are among major risk factors [11–13]. Requiring workers to follow procedures and practices related to infection control, injury prevention and the use of protective equipment can significantly reduce infections and needlestick injury [14, 15].

The aim of the current study is to investigate self-reported frequency of occupational infection and needlestick injury among the clinical laboratory workers in Al- Madinah. In addition, factors that are associated with these incidences were also examined.

AL-Madinah city, the second holiest site in Islam after Mecca, receives more than 10 million pilgrims each year, who come from all the world. The city provides health services to pilgrims and residents through 10 major hospitals and several medical centers. The considerable diversity of patients and heavy load highlight the importance of adopting good practices and safety protection measures to limit the spread of diseases in the city. Therefore, this study was designed to examine the self-reported occurrence of needlestick injuries, safety practices (i.e. recapping), and occupational infection among laboratory workers in Al-Madinah. The results of the current study can be used for interventions that target the enhancement of biosafety measures among Al-Madinah clinical laboratory workers.

## Methods

### Study participants

A survey-based study design was adapted to investigate the incidence and factors associated with needlestick injury and occupational infection among clinical laboratory workers in Al-Madinah city. Al-Madinah is the second holy city after Mecca in Saudi Arabia that host the Prophet’s Mosque. According to the Statistics Directorate, the population of Al-Madinah is estimated to be close to 1.5 million. The city receives more than 10 million pilgrims each year who came from most world countries.

Clinical laboratory staff from the majority of Al-Madinah clinics (eight private and ten governmental) was invited to be part of the study. Details about the purpose of the study and assurance of confidentiality were presented to participants as part of the recruitment procedure. About 405 participants were invited to fill out the questionnaire among which 234 agreed to participate (58%). The questionnaire was anonymous and self-administered and required about 5–8 min to fill out. This anonymity was a requirement that ensured no possible risks for the participants. To ensure confidentiality, the research team has removed the IP addresses from the data spreadsheet after completion of the recruitment process. The study was approved by the Institutional Review Board of the Faculty of Applied Medical Sciences (ID number: MLT 2016–23).

### Study instrument

The questionnaire was prepared from previous studies that examined needlestick injury, occupation safety and factors associated with their incidence [16–18]. The questionnaire comprised of 20 items that were presented with a choice of answers. The instrument was subjected to several revisions after comments were received from colleagues at the Department of Medical Laboratory Sciences and a pilot study that involved 20 staff from diagnostic clinical labs. The questionnaire was divided into three parts. The first part gathered information about participants’ age, gender, experience, prior training on biosafety, specialty, academic degree and place of work. The second part focused on needlestick injury and related behaviors such as covering needle after use (re-sheathing or re-capping). In this part, the participants were asked if they have experienced a needlestick injury during their career period. In addition, the participant was asked about frequency of re-capping needles after use. The third part focused on occupational infection and knowledge about disinfection procedures and infection routes. Participants were asked if they have experienced an occupational infection, which was defined as acquiring bacterial or viral infection from work place during their career period. Participants filled the questionnaire electronically using google forms.

### Statistical analysis

The SPSS software was used to analyze the data, which was presented as frequencies and number of participants in each category. Chi square test, Fisher Exact test and odd ratios with 95% confidence intervals were used to correlate demographic variables with needlestick and occupational infection. The *P* value of significance was set at 0.05 threshold.

## Results

A total of 234 medical laboratory workers was recruited to participate in the study. The majority (Table 1) of participants were young (57.7%, age range: 18–30), males (62.8%), married (55.6%), belong to governmental clinics (56.4%), bachelor degree holder (71.4%), have a specialty in clinical laboratory sciences (74.4%), works as technicians (60.3%) and received training on laboratory safety (65%). About 40% have less than 3 years of experience, whereas 27.4% have between 4 and 6 years of experience (Table 1). The sample is well distributed according different branches of laboratory sciences that include clinical chemistry (30.3%), hematology (42.3%), histology (11.5%) and microbiology/immunology (15.8%). All participants were vaccinated against HBV as this a requirement by health law in Saudi Arabia before employment in medical laboratories.

**Table 1:** General characteristics of participants

Variable	Category	Number of subjects	Percentage
Age	18–30	135	57.7%
	31–40	70	29.9%
	> 40	29	12.4%
Gender	Male	147	62.8%
	Female	87	37.2%
Social position	Married	130	55.6%
	Single	96	41.0%
	Divorced/widowed	8	3.4%
Place of work	Governmental clinics	132	56.4%
	Private clinics	102	43.6%
Academic degree	College degrez	30	12.8%
	Bachelor degree	167	71.4%
	Graduate degree	37	15.8%
Academic Field	Laboratory Sciences	174	74.4%
	Applied Biology	25	10.7%
	Health Science	19	8.1%
	Others	16	6.8%
Assigned work	Clinical chemistry	71	30.3%
	Hematology	99	42.3%
	Histology/pathology	27	11.5%
	Microbiology/ Immunology	37	15.8%
Years of experience	≤ 3	91	38.9%
	4–6	64	27.4%
	7–10	50	21.4%
	> 10	28	12.0%
Position	Residency	50	21.4%
	Technician	141	60.3%
	Lab director	18	7.7%
	Consultant	25	10.7%
Training on Biosafety	Yes	152	65.0%
	No	82	35.0%

The results showed that about 24% of the sample had experienced a needlestick injury. The results showed that the needlestick injury was associated with private clinics (*P* < 0.05) and lack of training on biosafety (Table 2). The participants were asked about capping needle directly after use. Approximately, 49% reported that they always do that, whereas 15% reported doing that most of the times (Table 3). Recapping needle after use, was associated with governmental clinics (*P* < 0.01), technician/residency staff (*P* < 0.01) and lack of training (*P* < 0.05, Table 3). Table 4 shows the incidence of occupational infection among participants. The incidence was about 33% and it was associated with college degrees (*P* < 0.05) and training on biosafety (*P* < 0.05, Table 4).

**Table 2:** Incidence of needle stick injuries among participants

Variable	Category	(Yes) Had needle stick injuries	(NO) Had needle stick injuries	Odd ratio	95% confidence interval	*P*. value
Age	18–30	32 (57.2%)	103 (57.9%)	–	–	
	31–40	18 (32.1%)	52 (29.2%)	1.123	0.60–2.09	0.714
	> 40	6 (10.7%)	23 (12.9%)	0.864	0.35–2.08	0.739
Gender	Male	39 (69.6%)	108 (60.7%)	–	–	
	Female	17 (30.4%)	70 (39.3%)	0.672	0.37–1.20	0.181
Social status	Married	34 (60.7%)	96 (53.9%)	–	–	
	Single	18 (32.1%)	78 (43.8%)	0.643	0.36–1.15	0.139
	Divorced/widowed	4 (7.1%)	4 (2.2%)	3.098	0.62–15.5	0.167
Place of work	Governmental clinics	25 (44.6%)	107 (60.1%)	–	–	
	Private clinics	31 (55.4%)	71 (39.9%)	1.833	1.04–3.21	0.034
Academic degree	College degree	8 (14.3%)	22 (12.4%)	–	–	
	Bachelor degree	38 (67.9%)	129 (72.5%)	0.798	0.34–1.84	0.598
	Graduate degree	10 (17.9%)	27 (15.2%)	1.020	0.36–2.88	0.957
Academic Field	Laboratory Sciences	40 (71.4%)	134 (75.3%)	–	–	
	Applied Biology	9 (16.1%)	16 (9.0%)	1.877	0.77–4.52	0.159
	Health Science	6 (10.7%)	13 (7.3%)	1.452	0.55–3.81	0.449
	Others	1 (1.8%)	15 (8.4%)	0.261	0.05–1.28	0.099
Assigned work	Clinical chemistry	15 (26.8%)	55 (30.9%)	–	–	
	Hematology	22 (39.3%)	77 (43.3%)	1.041	0.53–2.04	0.906
	Histology/pathology	8 (14.3%)	19 (10.7%)	1.460	0.56–3.75	0.431
	Microbiology/Immunology	10 (17.9%)	27 (15.2%)	1.377	0.58–3.24	0.464
Years of experience	≤ 3	16 (28.6%)	75 (42.1%)	–	–	
	4–6	18 (32.1%)	46 (25.8%)	1.782	0.88–3.59	0.106
	7–10	17 (30.4%)	33 (18.5%)	2.286	0.92–4.81	0.062
	> 10	4 (7.1%)	24 (13.5%)	0.724	0.26–2.01	0.536
Position	Residency	14 (25.0%)	36 (20.2%)	–	–	
	Technician	32 (57.1%)	109 (61.2%)	0.747	0.37–1.49	0.408
	Lab director	7 (12.5%)	(6.2%)11	1.733	0.56–5.37	0.341
	Consultant	3 (5.4%)	22 (12.4%)	0.333	0.10–1.10	0.072
Training on Biosafety	Yes	29 (51.8%)	123 (69.1%)	–	–	
	No	(48.2%) 27	(30.9%) 55	2.054	1.15–3.66	0.014

**Table 3:** Covering needle directly after use as reported by participants expressed as number of participants (%)

Variable	Category	Always	Most Times	Neutral	Sometimes	Never	*P*. value
Age	18–30	76 (61.3)	21 (61.8)	26 (48.1)	6 (50.0)	6 (60.0)	0.770
	31–40	35 (28.2)	8 (23.5)	18 (33.3)	6 (50.0)	3 (30.0)	
	> 40	13 (10.6)	5 (14.7)	9 (16.7)	0 (0.0)	1 (10.0)	
Gender	Male	80 (64.55)	18 (52.9)	36 (66.7)	8 (66.7)	5 (50.0)	0.610
	Female	44 (35.5)	16 (47.1)	18 (33.3)	4 (33.3)	5 (50.0)	
Social status	Married	73 (58.9)	18 (52.9)	27 (50.0)	7 (58.3)	5 (50.0)	0.804
	Single	46 (37.1)	16 (47.1)	24 (44.4)	5 (41.7)	5 (50.0)	
	Divorced/widowed	5 (4.0)	0 (0.0)	3 (5.6)	0 (0.0)	0 (0.0)	
Place of work	Governmental clinics	79 (63.7)	18 (52.9)	21 (38.9)	5 (41.7)	9 (90.0)	0.004
	Private clinics	45 (36.3)	16 (47.1)	33 (61.1)	7 (58.3)	1 (10.0)	
Academic degree	College degree	16 (12.9)	7 (20.6)	4 (7.4)	3 (25.0)	0 (0.0)	0.066
	Bachelor degree	88 (71.0)	26 (76.5)	41 (75.9)	6 (50.0)	6 (60.0)	
	Graduate degree	20 (16.1)	1 (2.9)	9 (16.7)	3 (25.8)	4 (40.0)	
Academic Field	Laboratory Sciences	87 (70.2)	24 (70.6)	46 (85.2)	10 (83.3)	7 (70.0)	0.631
	Applied Biology	14 (11.3)	4 (11.8)	4 (7.4)	1 (8.3)	2 (20.0)	
	Health Science	10 (8.1)	4 (11.8)	3 (5.6)	1 (8.3)	1 (10.0)	
	Others	13 (10.5)	2 (5.9)	1 (1.9)	0 (0.0)	0 (0.0)	
Assigned work	Clinical chemistry	37 (29.8)	9 (26.5)	15 (27.8)	7 (58.3)	3 (30.0)	0.658
	Hematology	50 (40.3)	17 (50.0)	25 (46.3)	3 (25.0)	4 (40.0)	
	Histology/pathology	14 (11.3)	6 (17.6)	4 (7.4)	2 (16.7)	1 (10.0)	
	Microbiology/ Immunology	23 (18.5)	2 (5.9)	10 (18.5)	0 (0.0)	2 (20.0)	
Years of experience	≤ 3	54 (43.5)	17 (50.0)	12 (22.2)	4 (33.3)	4 (40.0)	0.365
	4–6	31 (25.0)	6 (17.6)	18 (33.3)	5 (41.7)	4 (40.0)	
	7–10	23 (18.5)	8 (23.5)	16 (29.6)	3 (25.0)	0 (0.0)	
	> 10	15 (12.1)	3 (8.8)	8 (14.8)	0 (0.0)	2 (20.0)	
Position	Residency	32 (25.8)	8 (23.5)	10 (18.5)	0 (0.0)	0 (0.0)	0.001
	Technician	74 (59.7)	19 (55.9)	37 (68.5)	6 (50.0)	5 (50.0)	
	Lab director	9 (7.3)	4 (11.8)	3 (5.6)	0 (0.0)	2 (20.0)	
	Consultant	9 (7.3)	3 (8.8)	4 (7.4)	6 (50.0)	3 (30.0)	
Training on biosafety	Yes	69 (55.6)	24 (70.6)	46 (85.2)	7 (58.3)	6 (60.0)	0.024
	No	55 (44.4)	10 (29.4)	8 (14.8)	5 (41.7)	4 (40.0)

**Table 4:** Incidence of occupational infection among participants

Variable	Category	Yes	No	Odd ratio	95% confidence interval	*P*. value
Age	18–30	47 (60.2%)	88 (56.4%)	–	–	
	31–40	23 (29.5%)	47 (30.1%)	0.933	0.50–1.74	0.828
	> 40	8 (10.3%)	21 (13.5%)	0.666	0.27–1.62	
Gender	Male	47 (60.2%)	100 (64.1%)	–	–	
	Female	31 (39.7%)	56 (35.9%)	1.185	0.66–2.09	0.560
Social status	Married	43 (55.1%)	87 (55.8%)	–	–	
	Single	29 (37.2%)	67 (42.9%)	0.876	0.49–1.55	0.652
	Divorced/widowed	6 (7.7%)	2 (1.3%)	7.12	0.84–59.86	0.070
Place of work	Governmental clinics	43 (55.1%)	89 (57.1%)	–	–	
	Private clinics	35 (44.9%)	67 (42.9%)	1.084	0.620–1.895	0.775
Academic degree	College degree	17 (21.8%)	13 (8.3%)	–	–	
	Bachelor degree	50 (64.1%)	117 (75.0%)	0.310	0.12–0.74	0.009
	Graduate degree	11 (14.1%)	26 (16.7%)	0.318	0.10–0.93	0.037
Academic Field	Laboratory Sciences	56 (71.8%)	118 (75.6%)	–	–	
	Applied Biology	12 (15.4%)	13 (8.3%)	1.97	0.79–4.94	0.144
	Health Science	4 (5.1%)	15 (9.6%)	0.527	0.17–1.61	0.264
	Others	6 (7.7%)	10 (6.4%)	1.206	0.41–3.49	0.729
Assigned work	Clinical chemistry	29 (37.2%)	42 (26.9%)	–	–	
	Hematology	28 (35.9%)	71 (45.5%)	0.571	0.29–1.10	0.096
	Histology/pathology	8 (10.3%)	19 (12.2%)	0.608	0.22–1.61	0.317
	Microbiology/ Immunology	13 (16.7%)	24 (15.4%)	0.827	0.35–1.94	0.662
Years of experience	≤ 3	33 (42.3%)	58 (37.2%)	–	–	
	4–6	18 (23.1%)	46 (29.5%)	0.675	0.33–1.36	0.272
	7–10	15 (19.2%)	35 (22.4%)	0.760	0.35–1.62	0.478
	> 10	11 (14.1%)	17 (10.9%)	1.23	0.48–3.10	0.656
Position	Residency	19 (24.4%)	31 (19.9%)	–	–	
	Technician	47 (60.3%)	94 (60.3%)	1.400	0.57–3.39	0.457
	Lab director	5 (6.4%)	13 (8.3%)	0.750	0.24–2.29	0.613
	Consultant	7 (9.0%)	18 (11.5%)	0.751	0.29–1.91	0.546
Training on Biosafety	Yes	42 (53.8%)	110 (70.5%)	–	–	
	No	36 (46.2%)	46 (29.5%)	2.085	1.16–3.74	0.013

**Fig. 1:**
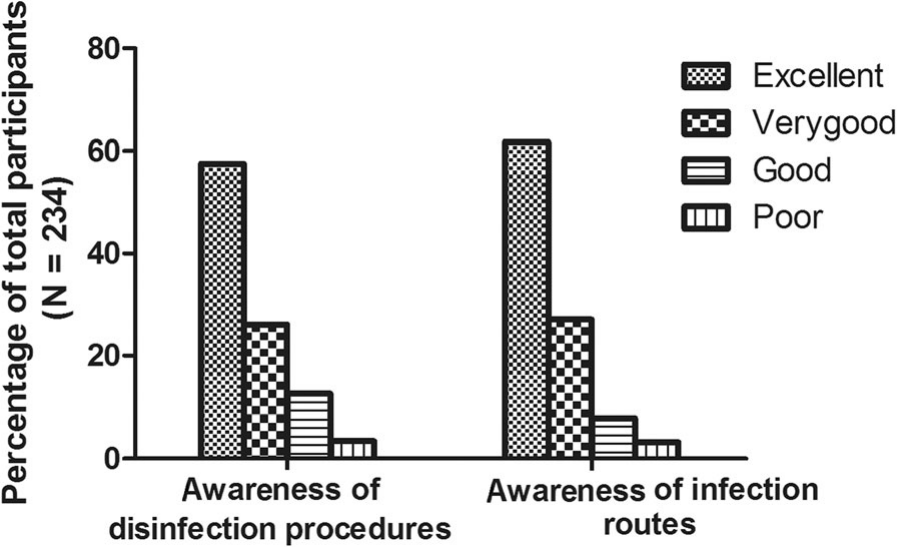
Awareness of participants about disinfection procedures and infection routes. A total of 234 participants were included in the study. More than 80% of study sample reported having excellent to very good awareness about disinfection procedures and infection routes

Figure 1 shows the awareness of participants about disinfection procedures and infection routes. The results showed that the majority of participants reported excellent to very good awareness levels (> 80%).

## Discussions

In this study, the incidence of occupational infection and needlestick injury among clinical laboratory workers in Al-Madinah city was investigated.

With respect to the self-reported frequency of needlestick injury, approximately 24% of the sample had such experience during their career period. This rate is comparable to what was reported in some previous studies [16–18]. For example, an incidence rate of 22.4% sharp injuries over a period of 12 months was reported in a cross-sectional study that was conducted in Dominican Republic [16]. In an Egyptian cross-sectional interview-based study, about 36% of participants reported exposure to at least 1 needlestick injury during the past 3 months [18]. However, higher frequencies (63–73%) were reported in cross-sectional survey-based studies that were conducted in Bosnia [19] and Afghanistan [20] and include the whole career period. Needlestick injuries reported in this study could be due to what was reported by the participants that they always (49%) recap needle after use, whereas 15% reported doing that most of the times. Recapping needle after use was associated technician/residency staff and lack of training. In a Poland study, 64% of respondents occasionally recap needles after injections [21]. In Morocco, 51% reported recapping needles after use [22]. In a review that was conducted by De Carli and colleagues [8], issues related to management of sharp disposals, needle recapping, and the transfer of sampled blood from syringes into tubes account for the majority of needlestick injuries. Thus, behavior of medical staff plays an important in sharp injuries [23]. Needlestick and sharp injuries can be prevented by applying educational and biosafety training programs and needle protective devices [24, 25]. The finding of the present study that needlestick injuries were strongly associated with the lack of training on biosafety and private clinics confirmed the importance of education in reducing sharp injuries in medical laboratories. Finally, the results showed that needledstick injuries were less frequent in governmental clinics and recapping was performed more frequently. Thus, additional factors seem to contribute to needlestick injury, such as workloads and adherence to safety guidelines that are expected to differ in governmental and private clinics. More studies are required to determine the exact factors that contribute to the observed high frequency of needlestick injury among Al-Madinah clinical laboratory workers.

The results showed that approximately 33% of participants experienced occupational infection during their career period. Previous studies have shown increased risk of clinical laboratory workers to diverse types of infection from their work places [26] that include blood borne pathogens (HBV, HCV, HIV), respiratory illnesses (MERS-CoV, influenza viruses, Tuberculosis) [27] and skin infections [28]. In a cross-sectional survey study that was conducted in clinics from 10 Moroccan cities, 58.9% of the subjects underwent at least one occupational blood exposure [22]. The results showed an association between occupational infection and college degree holders and training on biosafety.

The results showed that > 80% of the sample reported very-good to excellent knowledge regarding infection routes and disinfection procedures. Thus, other factors apart from education are likely to play a role in determining incorrect behaviors such as the adherence to infection control guidelines. However, the association between needlestick injury and occupational infection with lack of training on biosafety highlights the importance of training in reducing such biohazards. Previous studies have pointed to the effectiveness of the adherence to infection control guidelines, use of injury prevention devices and biosafety educational programs in the prevention of occupational infection and injury [29, 30].

In this cross-sectional study, we asked the participants if they have ever experienced needlestick injury or occupational infection. To have a better assessment of the current situation, conduction of a longitudinal study is strongly recommended where the incidence of such biohazards can be accurately measured. Inclusion of more questions in the assessment such as how often the participants perform phlebotomy and whether they use needlestick prevention devices are strongly recommended. Other limitations include the validity of key measures such as recall bias and social desirability related to recapping practices, selection bias and the data were not adjusted for confounder factors.

In conclusion, the frequency occupational infection and needlestick injury among clinical laboratory workers in Al-Madinah was relatively high as self-reported by participants. Strict implementation of biohazard guidelines in the health care settings and the use of needlestick prevention devices are recommended to reduce the risk of occupational health infections.
